# Feasibility of FnNO measurement in preschoolers and development of predictive models

**DOI:** 10.3389/fped.2026.1891740

**Published:** 2026-09-21

**Authors:** Yue Wang, Qianqian Lin, Haoyu Wang, Yanchun Li

**Affiliations:** Department of Pediatric Respiratory Medicine, Children's Medical Center, The First Hospital of Jilin University, Changchun, Jilin, China

**Keywords:** children, compliance, feasibility, FnNO, predictive model

## Abstract

**Background:**

Fractional nasal exhaled nitric oxide (FnNO) is a valuable biomarker of upper airway mucosal inflammation, but its clinical application in preschoolers is limited by uncertain compliance and a lack of reference data. This study aimed to evaluate the feasibility of standardized FnNO measurement in children aged 3–6 years, identify independent predictors of test cooperation and first-attempt success to build exploratory predictive models, and provide preliminary distribution estimates of FnNO in healthy preschoolers.

**Methods:**

A total of 148 preschoolers aged 3–6 years were enrolled. Test completion and first-attempt success rates were recorded. Multivariable logistic regression was used to identify independent predictors of compliance. Exploratory predictive models were developed and evaluated using receiver operating characteristic (ROC) analysis with internal bootstrap validation. FnNO distribution characteristics were analyzed in both the general cohort and a healthy subgroup.

**Results:**

The overall test completion rate was 82.4% (122/148), and the first-attempt success rate was 76.2% (77/101). Multivariable analysis demonstrated that female sex (OR = 4.853, *P* = 0.004) and greater height (OR = 1.216, *P* = 0.000) were independent predictors of test completion. Age was the sole independent predictor of first-attempt success (OR = 2.299, *P* = 0.001). The predictive model for test completion (sex and height) achieved an area under the curve (AUC) of 0.870, while the first-attempt success model (age and BMI) yielded an AUC of 0.749. The median FnNO level was 315.50 ppb (IQR: 168.00–410.00 ppb) across the overall cohort and 324.00 ppb (IQR: 221.00–378.50 ppb) in the healthy subgroup (*n* = 81).

**Conclusions:**

Standardized FnNO measurement is feasible in preschool children. Height, sex, and age are key factors influencing test cooperation. The proposed predictive models offer exploratory statistical tools for population stratification in research contexts, though prospective clinical validation remains necessary. This study establishes preliminary reference estimates for FnNO in Chinese preschoolers.

## 1 Introduction

Fractional nasal exhaled nitric oxide (FnNO) is a non-invasive, safe, and reproducible biomarker that directly reflects mucosal inflammation of the upper airways. Nitric oxide is synthesized by nitric oxide synthases in the respiratory mucosa, playing a central role in immune defense, ciliary motility, and mucus regulation (1, 2). Consequently, FnNO serves as a quantitative marker for sino-nasal inflammation and has established clinical utility in conditions such as allergic rhinitis, chronic rhinosinusitis, primary ciliary dyskinesia, and cystic fibrosis (3–7).

Despite its clinical value in school-aged children, data regarding FnNO applications in preschool-aged children (3–6 years) remain scarce. Most prior studies have focused on children older than 6 years or utilized tidal breathing techniques in younger infants, which differ fundamentally from standardized exhalation protocols (8). Standardized FnNO measurement requires controlled exhalation flow and sustained effort-maneuvers that pose behavioral and cognitive challenges for preschoolers. Poor cooperation frequently leads to invalid or uninterpretable test results. Furthermore, the physiological and neurodevelopmental determinants (such as age, height, sex, and weight) of test compliance in this age group are not fully understood, and validated tools to predict patient cooperation are lacking.

To address these knowledge gaps, this study evaluated the feasibility and success rates of standardized FnNO measurements in a cohort of preschoolers aged 3–6 years. The primary objective was to identify independent predictors of test completion and first-attempt success to develop exploratory predictive models. The secondary objective was to provide preliminary distribution estimates of FnNO levels in a healthy subset of preschool children.

## 2 Materials and methods

### 2.1 Study subjects

Preschool children aged 3–6 years were enrolled from two kindergartens in Changchun, Jilin Province, China, between July 2024 and July 2025. A total of 148 children (68 boys and 80 girls) participated. The cohort included 15 from the junior class, 36 from the middle class, 53 from the senior class, and 44 from the preschool class.

Inclusion Criteria: (1) Age 3 to 6 years; (2) Possess basic verbal communication skills; (3) With informed consent from the guardians.

Exclusion Criteria:

(1) Use of intranasal corticosteroids or antihistamines within 3 days prior to testing;
(2) Engagement in vigorous exercise, completion of pulmonary function tests, or failure to meet pre-test fasting requirements (water permitted) within 1 h prior to testing;
(3) Presence of organic diseases affecting major organs such as the heart, liver, or kidneys.

Healthy Subgroup Screening Criteria:

(1) No upper or lower respiratory tract infection or related symptoms within 2 weeks prior to enrollment;
(2) No history of diagnosed allergic diseases (e.g., allergic rhinitis, asthma, or atopic dermatitis) (8, 9).

The flowchart for participant selection in the experiment is presented in Figure 1.

**Figure 1:**
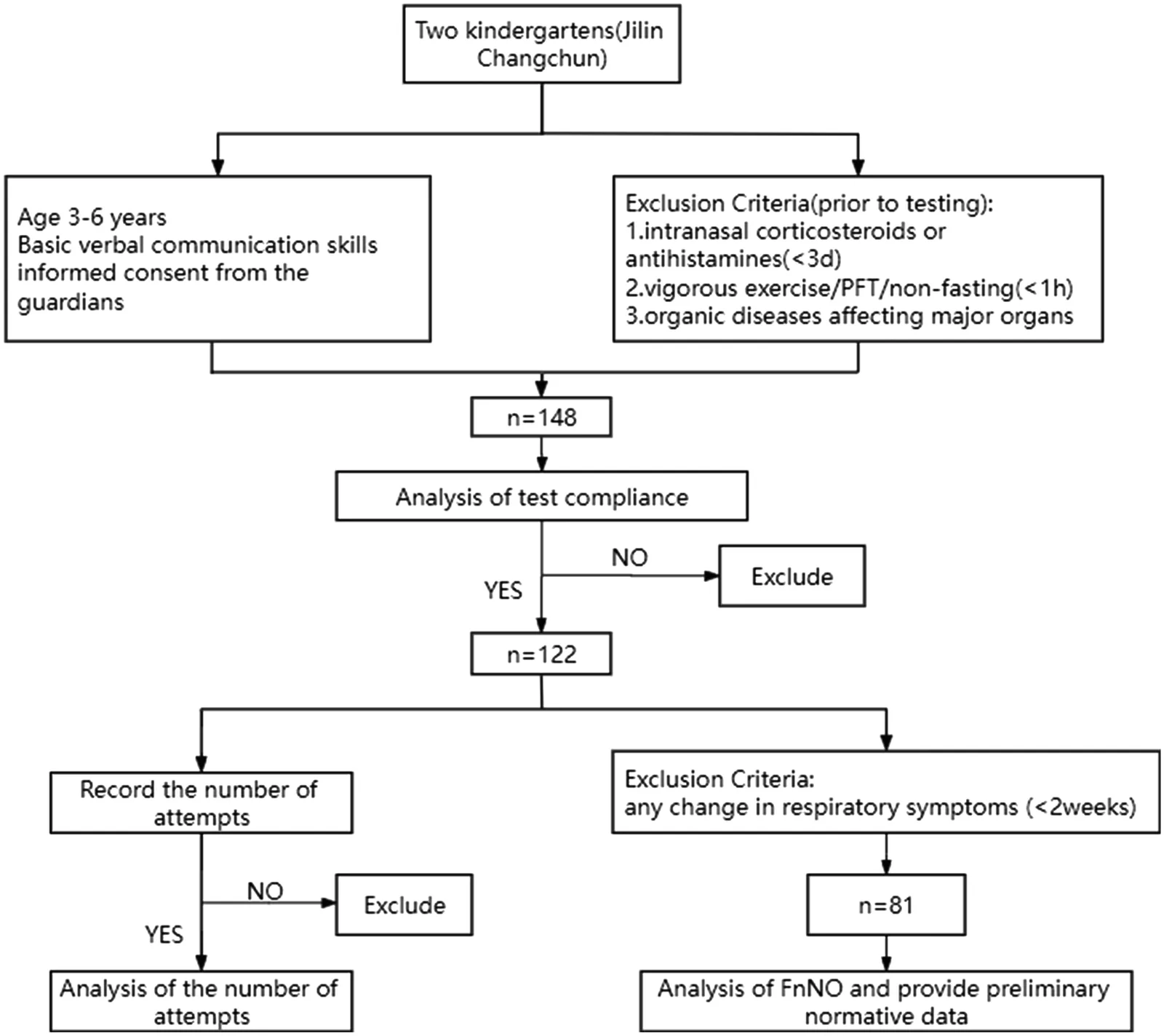
Study flow ndiagram. Flowchart illustrating participant recruitment, exclusion criteria, test compliance evaluation, first-attempt success analysis, and the selection of the healthy preschool subgroup for preliminary normative FnNO analysis.

### 2.2 Measurement equipment

FnNO measurements were conducted using a Sunvou-CA2122 breath analyzer (Wuxi Shangwo Medical Electronic Co., Ltd., Wuxi, China). The device utilizes nano-scale electrochemical gas sensor technology, featuring a detection range of 0–3,000 ppb and a measurement accuracy of ±3%.

### 2.3 Measurement protocol

Prior to testing, children gently blew their noses to clear nasal secretions and ensure upper airway patency. FnNO was measured using a standardized nasal exhalation technique: an olive-shaped nasal probe was inserted to form an airtight seal in one nostril while the contralateral nostril remained unoccluded. To ensure soft palate closure and prevent oral air leakage, the child blew into a target whistle held in the mouth, maintaining a steady exhalation guided by visual feedback on the analyzer screen (8, 10).

The sampling flow rate was automatically maintained at 10 mL/s, with a required minimum exhalation duration of ≥6 s (11). Each attempt was classified as successful or failed, and the cumulative number of attempts was recorded. To minimize procedure-induced anxiety and inter-operator variability, a single trained nurse conducted all sessions. Prior to formal testing, each child underwent a 2- to 3-min standardized training session involving demonstration and practice of the breathing technique. During formal testing, a maximum of two failed attempts was permitted before classifying the subject as non-compliant, and no repeated measurements were conducted for the same child on separate days.

### 2.4 Statistical analysis

Statistical analyses were performed using SPSS 26.0 software. Categorical variables were presented as counts (percentage) [*n* (%)] and were compared using the Chi-square (*χ*^2^) test. Quantitative variables were tested for normality. Non-normally distributed variables were expressed as median (interquartile range) [M (P25, P75)] and compared using non-parametric tests. Normally distributed variables were expressed as mean ± standard deviation ($\bar{x}\pm s$) and compared using *t*-tests.

Variables with *P* < 0.05 in univariate analysis were entered into a multivariable logistic regression model (enter method) after collinearity screening to identify independent influencing factors. Multicollinearity was defined as an absolute correlation coefficient |*r*| ≥ 0.70 and a Variance Inflation Factor (VIF) > 5.0. Based on these factors, prediction models for test completion and first-attempt success were constructed using the rms package. Nomograms were plotted, and receiver operating characteristic (ROC) curves were generated. The area under the curve (AUC) was calculated to evaluate model discrimination, with an AUC > 0.7 indicating good predictive performance. Internal validation of the predictive models was performed using a bootstrapping resampling approach (1,000 repetitions) to evaluate model calibration and discrimination. ROC curve analysis was accomplished through the use of the pROC package.

All statistical tests were two-tailed, with a significance level of *α* = 0.05. *P* < 0.05 indicated statistically significant differences.

### 2.5 Ethics approval and consent

The study protocol was approved by the Ethics Committee of The First Hospital of Jilin University (Approval No. 24K041-001).

## 3 Results

### 3.1 Baseline characteristics

The baseline characteristics of the 148 enrolled children are summarized in Table 1.

**Table 1:** Clinical characteristics of the study population.

Age (years)/ Variable	Total (*n* = 148)	3 (*n* = 23)	4 (*n* = 37)	5 (*n* = 46)	6 (*n* = 42)
Sex (male, *n* %)	68 (45.95%)	11 (47.83%)	18 (48.65%)	21 (45.65%)	18 (42.86%)
Weight (kg)	21.03 ± 6.86	15.76 ± 2.47	19.03 ± 5.80	21.11 ± 4.99	25.59 ± 8.25
Height (cm)	112.46 ± 8.39	101.46 ± 3.58	108.07 ± 5.84	114.19 ± 5.56	120.45 ± 5.26
BMI (kg/m^2^)	16.4 ± 3.98	15.28 ± 2.13	16.17 ± 4.00	16.14 ± 3.32	17.51 ± 5.11
Age (years)	4.72 ± 1.04	–	–	–	–

### 3.2 Test compliance and predictive nomogram

#### 3.2.1 Overall completion rate

Among the 148 children, 122 successfully completed the FnNO measurement, while 26 did not. The overall completion rate of the tests was 82.4%.

#### 3.2.2 Univariate analysis of compliance

Univariate analysis demonstrated significant differences between the compliant and non-compliant groups with respect to sex (*P* = 0.009), class level (*P* < 0.001), age (*P* < 0.001), height (*P* < 0.001), and weight (*P* < 0.001). BMI did not differ significantly between the two groups (*P* = 0.401; Table 2).

**Table 2:** Univariate analysis of factors influencing the completion of FnNO testing [*n* (%), M (P25, P75)].

Variable	Whether to complete testing		Statistics	*P* value
	Yes (*n* = 122)	No (*n* = 26)		
Sex			*χ*^2^ = 6.886	0.009
Male	50 (73.53)	18 (26.47)		
Female	72 (90.00)	8 (10.00)		
Class level			*χ*^2^ = 23.309	<0.001
Junior class	9 (60.0)	6 (40.0)		
Middle class	23 (63.89)	13 (36.11)		
Senior class	47 (88.68)	6 (11.32)		
Preschool class	43 (97.73)	1 (2.27)		
Age (years)			*χ*^2^ = 25.791	<0.001
3	13 (56.52)	10 (43.48)		
4	28 (75.68)	9 (24.32)		
5	39 (84.78)	7 (15.22)		
6	42 (100.00)	0 (0.00)		
Height (cm)	115.00 (107.80,120.00)	104.00 (100.00,107.30)	*Z* = 5.216	<0.001
Weight (kg)	20.00 (17.35,24.00)	16.60 (14.88,17.85)	*Z* = −4.210	<0.001
BMI	15.37 (14.29,17.65)	15.22 (13.98,16.12)	*Z* = −0.839	0.401

#### 3.2.3 Multivariable logistic regression analysis of test completion

Variables that showed statistical significance (*P* < 0.05) in the univariate analysis-sex, age, class level, height, and weight-were first assessed for collinearity. Variables exhibiting significant multicollinearity were excluded prior to fitting the final multivariable model. Multivariable logistic regression identified female sex (OR = 4.853, 95% CI: 1.669–14.113, *P* = 0.004) and greater height (OR = 1.216, 95% CI: 1.120–1.321, *P* < 0.001) as independent predictors for successful test completion (Table 3 and Supplementary Table S1).

**Table 3:** Multivariate logistic regression analysis of factors influencing the completion of FnNO testing.

Variable	Assignment	B	SE	Wald *χ*^2^	*P*	OR	95%CI
Sex	male = 0, female = 1	1.623	0.553	8.602	0.003	5.067	1.713∼14.987
Age (years)	Continuous variable	0.024	0.414	0.003	0.953	0.976	0.434∼2.197
Weight (kg)	Continuous variable	0.040	0.079	0.250	0.617	1.041	0.890∼1.216
Height (cm)	Continuous variable	0.181	0.073	6.203	0.013	1.198	1.039∼1.382
Constant		19.545	6.094	10.286	0.001	0.000	

#### 3.2.4 Development of a prediction model for test compliance

A final prediction model for FnNO test compliance was developed based on the two independent influencing factors: sex and height. The nomogram prediction model yielded an AUC of 0.870 (95% CI: 0.804–0.935), with a sensitivity of 77.0% and a specificity of 88.5%. The optimal height cutoff value determined by the Youden index was 108.25 cm (AUC = 0.826, sensitivity = 73.8%, specificity = 80.8%). Internal validation via 1,000 bootstrap resamples confirmed robust discrimination, yielding a mean bootstrap AUC of 0.870 (SD = 0.034, 95% CI: 0.799–0.928) (Figure 2). The clinical nomogram based on this model is illustrated in Figure 3.

**Figure 2:**
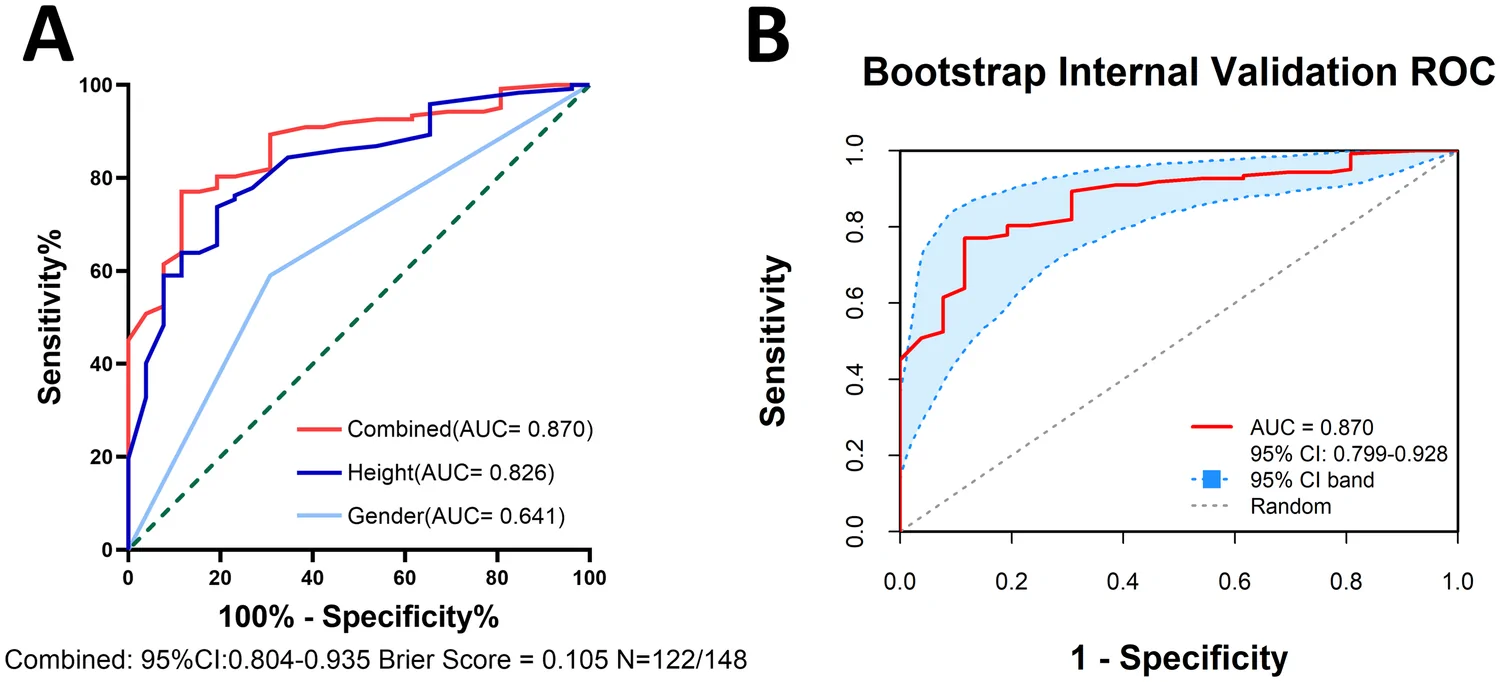
Predictive model for overall test compliance in preschool children. **(A)** ROC curves of height, sex, and the combined predictive model for test compliance. The combined model achieved an AUC of 0.870. **(B)** Bootstrap internal validation ROC curves for the prediction model.

**Figure 3:**
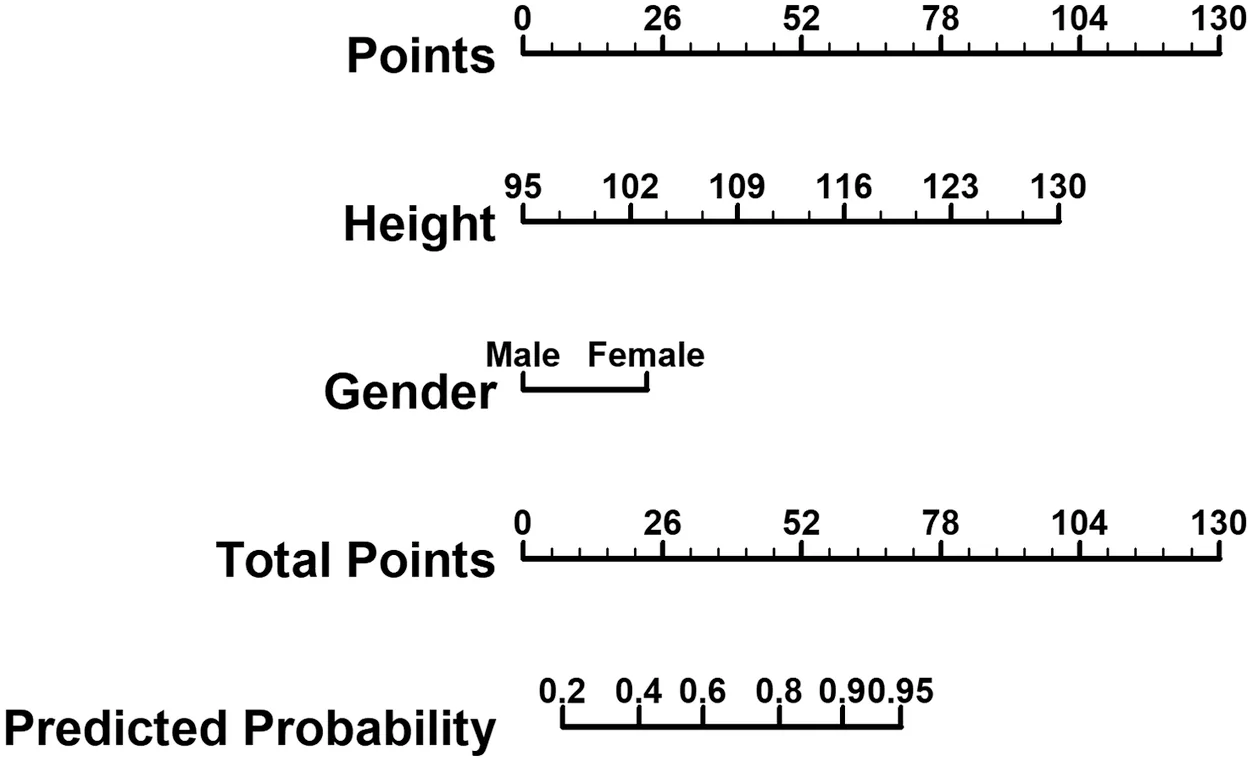
Nomogram incorporating sex and height to estimate the individual probability of test compliance.

### 3.3 First-attempt success and predictive nomogram

#### 3.3.1 Overall first-attempt success rate

Among the 122 compliant children, 101 had complete documentation of the number of attempts. Of these, 77 children (76.2%) successfully completed the test on the first attempt, while 24 required two or more attempts. The overall first-attempt success rate was 76.2%.

#### 3.3.2 Univariate analysis of first-attempt success

Univariate analysis showed that class level (*P* < 0.001), age (*P* = 0.003), weight (*P* = 0.015), and BMI (*P* = 0.040) were significantly associated with first-attempt success. Sex and height showed no significant association (*P* > 0.05; Table 4).

**Table 4:** Univariate analysis of factors influencing the first-attempt success rate of FnNO testing [*n* (%), M (P25, P75)].

Variable	Number of completed tests		Statistics	P
	first-attempt (*n* = 77)	multiple-attempt (*n* = 24)		
Sex			*χ*^2^ = 0.137	0.712
Male	32 (74.42)	11 (25.58)		
Female	45 (77.59)	13 (22.41)		
Class level			*χ*^2^ = 17.885	<0.001
Small class	2 (22.22)	7 (77.78)		
Middle class	10 (90.91)	1 (9.09)		
Large class	27 (71.05)	11 (28.95)		
Preschool class	38 (88.37)	5 (11.63)		
Age (years)			*χ*^2^ = 13.902	0.003
3	3 (30.00)	7 (70.00)		
4	11 (68.75)	5 (31.25)		
5	26 (78.79)	7 (21.21)		
6	37 (88.10)	5 (11.90)		
Height (cm)	117.00 (111.50,121.00)	115.00 (102.00,120.00)	*Z* = −1.652	0.098
Weight (kg)	21.00 (18.00,25.50)	17.75 (15.25,22.00)	*Z* = −2.433	0.015
BMI	15.61 (14.18,18.17)	14.47 (13.18,15.97)	*Z* = −2.051	0.040

#### 3.3.3 Multivariable analysis of first-attempt success

Multivariable logistic regression analysis revealed that age was the sole independent predictor of first-attempt success (OR = 2.299, 95% CI: 1.402–3.771, *P* = 0.001). For each additional year of age, the odds of succeeding on the first attempt increased by 129.9% (Supplementary Table S2).

#### 3.3.4 Development of a prediction model for first-attempt success

Based on the independent influencing factor of age, BMI was retained to develop a combined prediction model (age + BMI). The ROC curve analysis revealed that the AUC for the combined model was 0.749 (95% CI: 0.635–0.864), which was higher than that of the age-only model (AUC = 0.711), with a sensitivity of 75.3% and a specificity of 66.7%. The optimal cutoff values for each variable, determined by ROC curve analysis, were age ≥4.5 years (AUC = 0.711, sensitivity 81.8%, specificity 50.0%) and BMI ≥ 14.82 kg/m^2^ (AUC = 0.639, sensitivity 63.6%, specificity 66.7%). Bootstrap validation (1,000 iterations) yielded a mean AUC of 0.753 (SD = 0.064, 95% CI: 0.622–0.867) (Figure 4). The nomogram for predicting first-attempt success is presented in Figure 5.

**Figure 4:**
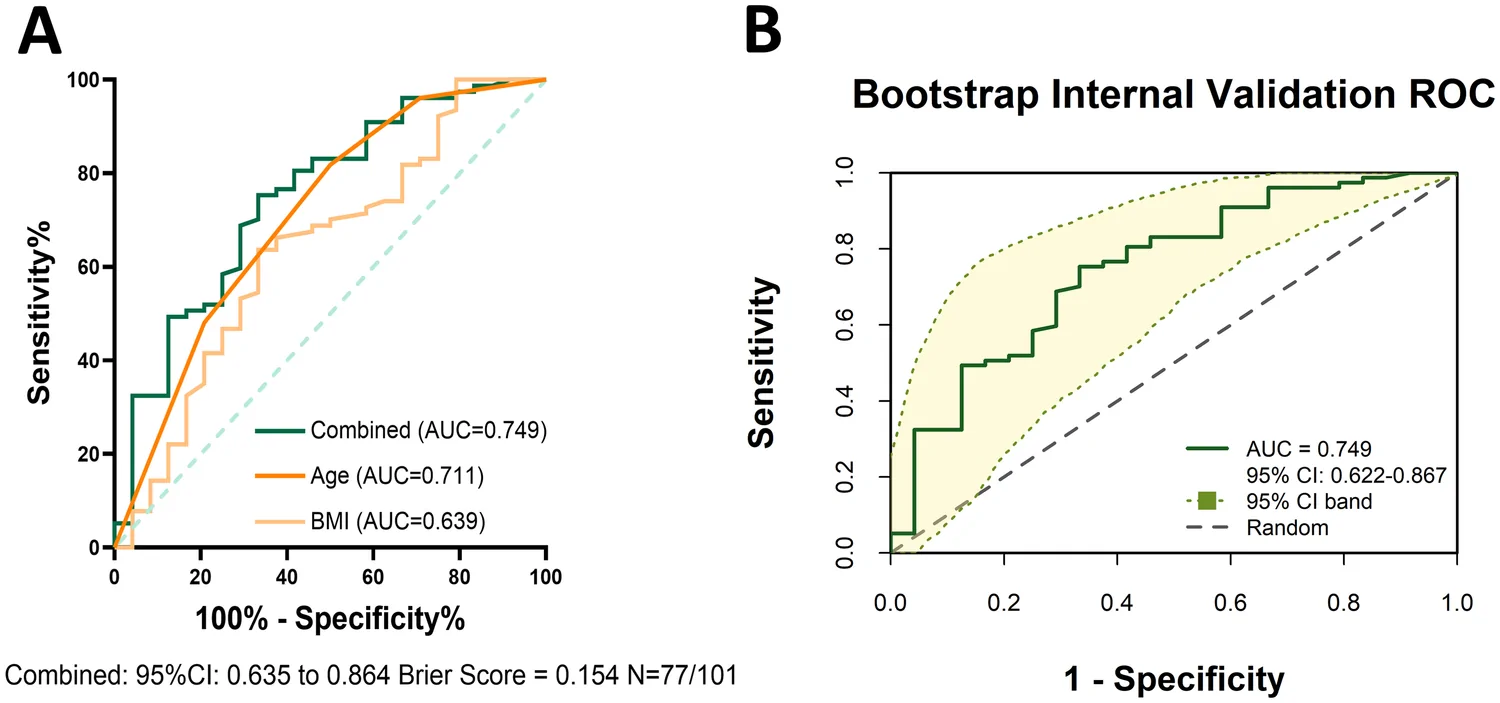
Predictive model for first-attempt success in FnNO testing. **(A)** ROC curves of age, BMI, and the combined model for predicting first-attempt success. The combined model achieved an AUC of 0.749. **(B)** Bootstrap internal validation ROC curves for the prediction model.

**Figure 5:**
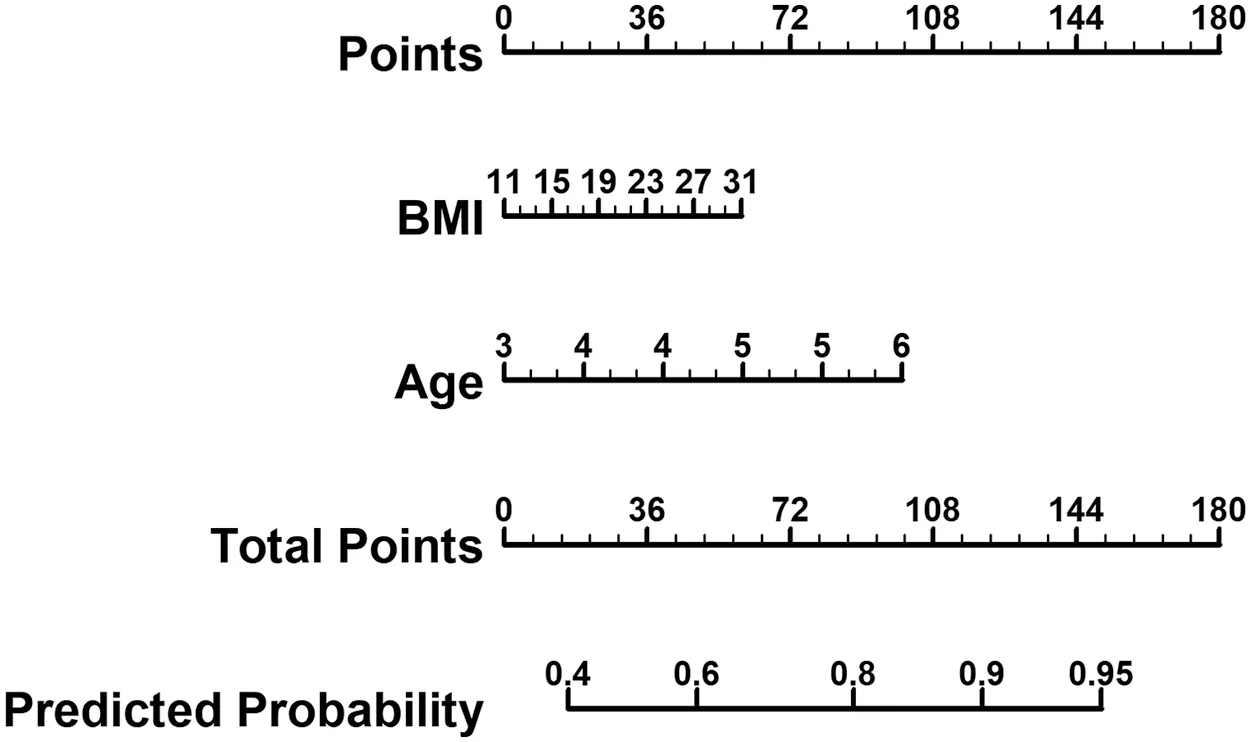
Nomogram incorporating age and BMI to predict the likelihood of succeeding on the first attempt.

### 3.4 Distribution of FnNO values

In the overall cohort (*N* = 122), FnNO values exhibited a non-normal, right-skewed distribution with a median of 315.50 ppb (168.00–410.00 ppb; Supplementary Table S3, Figure S1). No significant differences were observed across age sub-groups (Kruskal–Wallis *H* = 6.492, *P* = 0.090).

In the healthy subgroup (*n* = 81), after rigorously excluding children with recent respiratory infections or allergic histories, the median FnNO level was 324.00 ppb (221.00–378.50 ppb; Supplementary Table S4, Figure S2). Comparison across different age groups for healthy children revealed no statistically significant differences (Kruskal–Wallis *H* = 1.842, *P* = 0.606).

## 4 Discussion

This study evaluated the feasibility of standardized FnNO measurement in preschool children aged 3–6 years, achieving an overall completion rate of 82.4% and a first-attempt success rate of 76.2%. Female sex and greater height were identified as independent predictors of test completion, while age independently predicted first-attempt success. Additionally, preliminary FnNO distribution estimates were established in a healthy subgroup of preschoolers (median: 324.00 ppb; P25–P75: 221.00–378.50 ppb).

### 4.1 Feasibility and determinants of test cooperation

Our findings indicate that with standardized operational guidance and a brief pre-test training session, the majority of preschoolers can successfully undergo FnNO testing. Previous studies using tidal breathing in children under 5 years old reported completion rates around 69% (12). Although direct comparison is limited due to methodological differences, our higher completion rate highlights the clinical viability of active exhalation maneuvers in 3–6-year-olds when accompanied by standardized coaching.

Multivariable regression analysis identified female sex and greater height independently predicted successful test completion. Specifically, each 1-cm increase in height was associated with a 21.6% increase in the odds of completing the test. Height serves as a surrogate indicator for thoracic development and lung capacity; taller children are better equipped to sustain a steady exhalation flow (10 mL/s for ≥6 s) without air leakage. Furthermore, girls demonstrated a significantly higher completion rate than boys (OR = 4.853). This is consistent with pediatric behavioral literature indicating that preschool-aged girls often exhibit superior neurodevelopmental maturity, sustained attention, and self-regulatory skills compared to age-matched boys (13).

Regarding first-attempt success, age emerged as the sole independent predictor (OR = 2.299 per year). This underlines the vital role of cognitive comprehension and emotional regulation in performing the maneuver correctly on the initial try. Although BMI was not an independent predictor, incorporating BMI into the predictive model slightly improved its discriminatory capacity (AUC increased from 0.711 to 0.749).

### 4.2 Clinical context and model validation

The predictive nomograms developed herein demonstrated good discriminatory performance (AUC = 0.870 for compliance; AUC = 0.749 for first-attempt success). Bootstrap validation confirmed model stability, with optimistic-corrected AUC = 0.864 for compliance and optimistic-corrected AUC = 0.727 for first-attempt success, indicating that the risk of model overfitting is relatively low. The narrow 95% CI for the compliance model (0.799–0.928) supports its statistical reliability in this population. In comparison, the 95% CI of the prediction model for first-attempt success is 0.622–0.867. While the wider confidence interval points to greater variability, its lower bound still exceeds 0.5, supporting the model's potential utility.

We emphasize that these nomograms are intended as exploratory statistical tools to assist researchers in stratifying patient populations and tailoring coaching intensity rather than rigid clinical screening rules. Prospective multi-center validation in broader clinical settings is recommended before integrating these models into routine practice.

### 4.3 Distribution characteristics of FnNO in healthy preschoolers

In our healthy preschool cohort (*n* = 81), the median FnNO level was 324.00 ppb (IQR: 221.00–378.50 ppb), with no statistically significant differences observed across individual age groups. Notable discrepancies were found between our preliminary estimates and previously published literature. Specifically, one previous study reported a mean FnNO value of 208 ppb in healthy children aged 6 months to 5 years (12), while another study observed a mean value of 186 ppb in healthy subjects aged 5–15 years (14). These variations in normal values across studies may be primarily attributed to differences in the age composition of the study populations as well as heterogeneous measurement techniques.

Regarding intra-cohort trends, this lack of significant age-dependent variation contrasts with the age-dependent increase reported in older children (6–18 years) (8). In preschoolers, stable FnNO levels may reflect a physiological balance between progressive sinus development (which increases NO production) and the high prevalence of transient adenoidal tissue hypertrophy (which physically impedes NO passage from the paranasal sinuses into the nasal cavity) (15).

### 4.4 Research limitations and prospects

Several limitations should be noted. First, participants were recruited from two kindergartens within a single geographic area, and symptomatic children requiring active medical therapy were excluded, limiting the overall generalizability of the findings to broader clinical populations. Second, while internal validation was performed using bootstrap resampling, external validation in an independent cohort is warranted. As all tests were performed by a single operator, formal inter-observer reproducibility was not assessed in this study. Future multi-center studies with larger sample sizes should aim to refine age-specific normative reference ranges and evaluate the impact of tailored behavioral guidance strategies on testing success.

## 5 Conclusion

Standardized FnNO measurement is feasible in children aged 3–6 years. Sex, height, and age are key determinants of test cooperation and first-attempt success. The proposed predictive models provide promising discriminatory performance for research stratification. This study establishes preliminary estimates of normal FnNO values in Chinese preschoolers, laying a foundation for future standardized clinical applications.

## Data Availability

The raw data supporting the conclusions of this article will be made available by the authors, without undue reservation.
