# Supplementary material for: Feasibility of FnNO measurement in preschoolers and development of predictive models
Source: Front Pediatr. 2026 Sep 21;14:1891740. doi: 10.3389/fped.2026.1891740 (PMC13635358; doi:10.3389/fped.2026.1891740)
Supplement: Supplementary file 1 [file Supplementaryfile1.docx]

Table S1.

Logistic regression analysis of the prediction model for test compliance

| Variable | Assignment | B | SE | Waldχ^2^ | P | OR | 95%CI |
| --- | --- | --- | --- | --- | --- | --- | --- |
| Height(cm) | continuous variable | 0.196 | 0.042 | 21.776 | 0.000 | 1.216 | 1.120~1.321 |
| Sex | male=0, female=1 | 1.580 | 0.545 | 8.412 | 0.004 | 4.853 | 1.669~14.113 |
| Constant | - | 20.507 | 4.541 | 20.397 | 0.000 | 0.000 |  |

Note: B, unstandardized regression coefficient; SE, standard error; OR, odds ratio; CI, confidence interval.

Table S2.

Multivariate logistic regression analysis of predictors for first-attempt success in FnNO testing

| Variable | Assignment | B | SE | Waldχ^2^ | P | OR | 95%CI |
| --- | --- | --- | --- | --- | --- | --- | --- |
| Age (years) | continuous variable | 0.832 | 0.252 | 10.874 | 0.001 | 2.299 | 1.402~3.771 |
| BMI (kg/m^2^) | continuous variable | 0.068 | 0.068 | 0.995 | 0.32 | 1.070 | 0.973~1.222 |
| Constant | - | 3.981 | 1.581 | 6.338 | 0.01 | 0.019 |  |

Note: B, unstandardized regression coefficient; SE, standard error; OR, odds ratio; CI, confidence interval.

Table S3.

Comparison of FnNO values in overall children across age groups [median (P25, P75), ppb]

| Age(years) | N | Median | P25, P75 |
| --- | --- | --- | --- |
| 3 | 13 | 265.00 | (64.00,361.00) |
| 4 | 28 | 281.50 | (118.00,368.50) |
| 5 | 39 | 276.00 | (104.00,414.00) |
| 6 | 42 | 351.00 | (261.00,465.00) |
| Total | 122 | 315.50 | (168.00,410.00) |

Note: Kruskal-Wallis H = 6.492, P = 0.090.

Table S4.

Comparison of FnNO values in healthy children across age groups [median (P25, P75), ppb]

| Age(years) | N | Median | P25, P75 |
| --- | --- | --- | --- |
| 3 | 9 | 324.00 | (102.50,368.00) |
| 4 | 18 | 320.50 | (222.50,438.00) |
| 5 | 27 | 278.00 | (163.00,380.00) |
| 6 | 27 | 340.00 | (259.00,410.00) |
| Total | 81 | 324.00 | (221.00,378.50) |

Note: Kruskal-Wallis H = 1.842, P = 0.606.

Figure S1. Frequency distribution of FnNO values in the overall cohort (N = 122).

Histogram with kernel density estimation curve illustrating the distribution of measured FnNO values across the overall preschool population (Mean ± SD: 309.83 ± 197.67 ppb).

Figure S2. Age-wise comparison of FnNO levels in healthy preschoolers (n = 81).

Violin plots with dashed lines representing the median and interquartile range (IQR). Kruskal-Wallis test revealed no statistically significant differences in FnNO levels across age groups (ns: not significant, P > 0.05), indicating a homogeneous distribution pattern across 3- to 6-year-olds.
